# High-Resolution Transperineal Ultrasound in Anorectal Malformations—Can We Replace the Distal Colostogram?

**DOI:** 10.1055/s-0042-1750027

**Published:** 2022-07-19

**Authors:** Francesca Palmisani, Wilfried Krois, Janina Patsch, Martin Metzelder, Carlos A. Reck-Burneo

**Affiliations:** 1 Department of Pediatric Surgery, Medical University of Vienna, Wien, Austria; 2 Department of Biomedical Imaging and Image-Guided Therapy, Medical University of Vienna, Wien, Austria

**Keywords:** anorectal malformations, ultrasound, diagnostics, reconstructive surgery

## Abstract

**Introduction** Anorectal malformations (ARM) affect 1 in 5,000 newborns with a wide range of defects. In the absence of a visible fistula, the diagnosis and classification of ARM require an augmented pressure distal colostogram. This procedure can be done after a diverting colostomy has been performed and implies exposing the child to radiation. We hypothesized that high-resolution transperineal ultrasound could correctly diagnose the type of ARM, thus sparing radiation exposure.

**Case Description** Four full-term male newborns with ARM and no visible anal opening were referred to our center for further management. A diverting descendostomy was performed in the first 48 hours of life in all cases. Prior to the reconstructive surgery, we performed a high-resolution transperineal ultrasound with 3D tomographic reconstruction of the perineal region to assess the urethra, the rectum, and a possible fistula. Findings were compared with a conventional augmented pressure distal colostogram. The image acquisition was fast and did not cause any additional distress to the children.

**Conclusion** In all cases the results of the distal colostogram nicely correlated with the high-resolution transperineal ultrasound with 3D tomographic reconstruction. In the future, we envision a time when it can potentially replace the distal colostogram in preoperative assessment of ARM with no distress and exposure to radiation.

## Introduction

Anorectal malformations (ARMs) consist of a wide spectrum of defects with an estimated incidence of 1:5,000 live births. 1 Management relies on the correct identification of the rectum's location and its relationships to the urinary tract and bony structures of the pelvis. 2 At present, the gold standard for diagnosis is the augmented-pressure distal colostogram which exposes patients to significant radiation.

We present a new technology called 3D tomographic ultrasound (tUS), that can potentially replace the distal colostogram without radiation exposure, which we tested on four newborn males with imperforated anus and no visible fistula. The technology employed (PIUR Imaging systems) connects a regular ultrasound transducer through digital video output (ex. HDMI) with an applied independent clip-on sensor device. During the sonography the two-dimensional images are transferred to the software (PIUR Infinity Workstation) in real time. The 3D picture is generated with a semi-automated segmentation process and can be visualized on any connected computer.

To our knowledge this is the first attempt to apply transperineal, high-resolution ultrasound with additional 3D tomographic diagnostics to patients with ARM prior to definitive corrective surgery.

## Case Series Report

Four full-term male newborns with imperforated anus were referred to our center at day 1 of life. As none presented signs of a perineal fistula, a diverting colostomy was performed in the first 48 hours. The descendostomy was performed according to standard with a separate mucous fistula to the distal colonic segment 3 and all patients underwent complete VACTERL screening. Complete clinical data of the four patients is illustrated in Table 1 .

**Table 1:** Clinical data of the reported cases

	Patient 1	Patient 2	Patient 3	Patient 4
Gestational age of birth (weeks)	36 ^6/7^	38 ^2/7^	39 ^0/7^	38 ^5/7^
Weight at birth	2910 g	3180 g	3240 g	2990 g
Visual appearance at birth	ARM with no visible fistula	ARM with no visible fistula	ARM with no visible fistula	ARM with no visible fistula
Meconium in the urine	No	No	Yes	Yes
Comorbidities	Hypospadia, hydronephrosis I right kidney with multiple dysplastic cysts, small VSD, ASD II	Hydronephrosis I-II left kidney	Hydronephrosis I-II right kidney	None
Age at colostomy (days)	2	2	2	1
Age at colostogram date (days)	16	8	6	8
Age at perineal US (days)	16	8	6	8
Diagnosis according to colostogram and perineal US	ARM with recto-bulbar fistula	ARM with recto-prostatic fistula	ARM with recto-bulbar fistula	ARM with recto-prostatic fistula
Age at PSARP (days)	58	50	45	87
Follow-up (weeks postop)	55	55	25	10

**Fig. 1:**
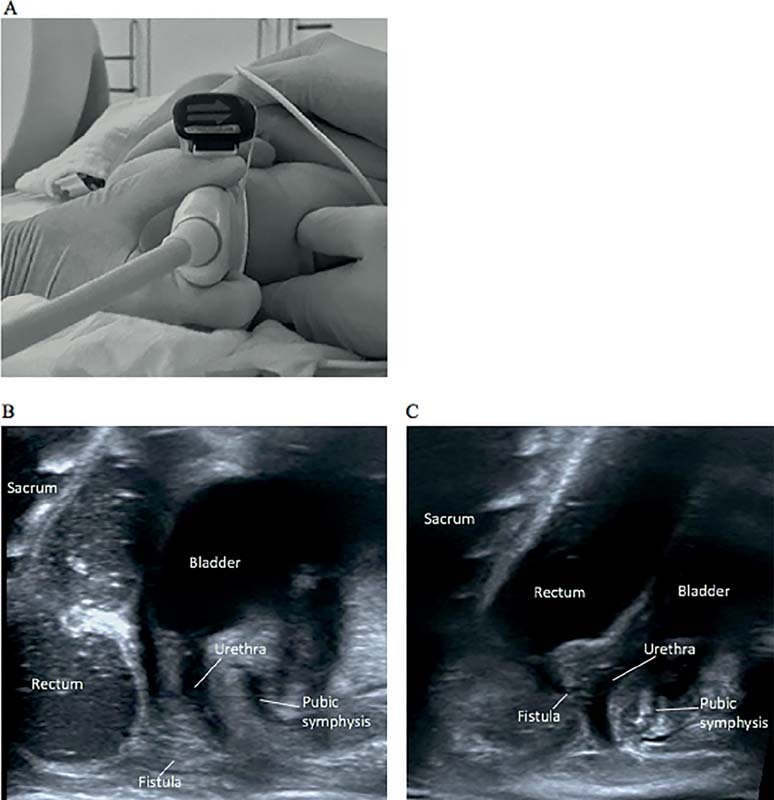
High resolution perineal ultrasound images: position of the linear transducer with PIUR tUS Infinity System add on mediansagittal at the center of the perineal body, patient supine held in lithotomy position, foley cathter in the mucous fistula. ( **A** ); recto-bulbar fistula ( **B** ); recto-prostatic fistula ( **C** ).

Preoperative assessment was performed through augmented pressure distal colostogram and high-resolution transperineal ultrasound with 3D tomographic reconstruction. The same ground principles of the standard colostogram were applied to the sonographic evaluation. 4 A Foley catheter was introduced in the mucous fistula and the injection of normal saline solution (0.9% NaCl) assured the dynamic visualization of the recto-urethral fistula. A high-resolution linear probe (14 Mhz transducer; Toshiba 14L5) with the PIUR tUS Infinity system add-on (Piur Imaging GmbH Vienna, Austria) was positioned on the perineum ( Fig. 1A ). After documentation of the anatomy in B-mode (including video loops), the add-on scan was performed by parallel probe sliding from left-to-right in a single acquisition (with video documentation). The distinction between recto-bulbar (two cases) and recto-prostatic fistulas (two cases) was possible by direct identification of the fistula relative to the rectum and the urethra, as well as indirect visualization of the prostate ( Fig. 1B , C ).

**Fig. 2:**
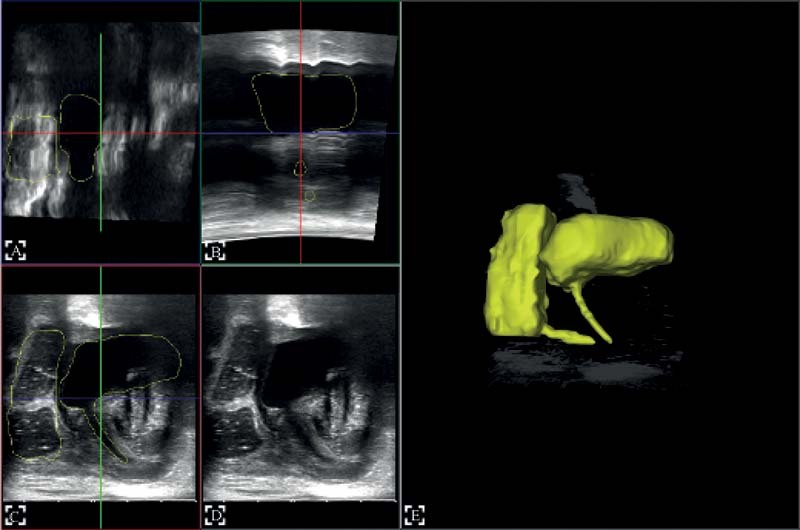
3D reconstruction process: on the left side respectively two coronal ( **A, B** ) and two sagittal cuts ( **C, D** ) created from the ultrasound acquisition, which are used to automatically reconstruct the structure, shown on the right side ( **E** ).

The 3D tomographic reconstruction was then automatically generated by the PIUR tUS Infinity system through threshold-based automatic segmentation of the images ( Fig. 2 ). Although the diagnosis was already apparent by high-resolution transperineal ultrasound in B-mode, the 3D reconstruction was found valuable for the planning of surgery, as well as for the illustration of the ARM to the parents. The images offered a better spatial visualization of the anatomy.

**Fig. 3:**
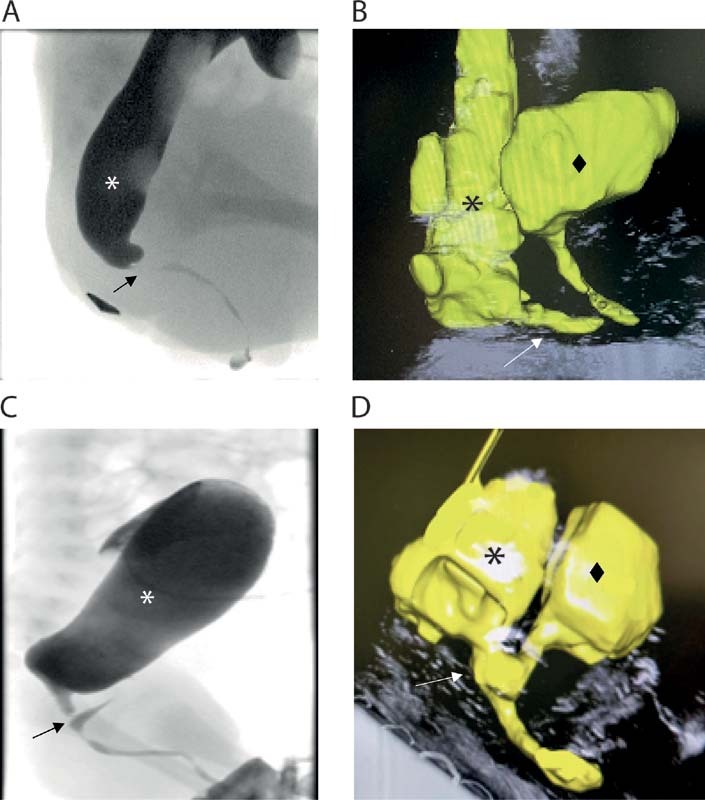
Comparison between distal colostogram ( **A, C** ) and the automatically 3D reconstructed structures, including bladder, urethra, and recto-urethral fistula, acquired from the linear ultrasound imaging ( **B, D** ) from the same patient. **A** and **B** show a recto-bulbar fistula, **C** and **D** a recto-prostatic fistula. The star indicates the rectum, the diamond shape of the bladder, and the arrow points to the recto-urethral fistula.

In all cases the results of the distal colostogram nicely correlated with the high-resolution transperineal US by B-mode and tUS ( Fig. 3A – D ). It is to be noted that the observers were not blinded to the results of the two investigations, but the diagnoses resulted concordant regardless of the order in which the two were performed. Indeed, the type of malformation identified was consistent both when the perineal tUS followed the distal colostogram and in the cases where it preceded it.

All the investigations were performed in the presence of the parents, to ease the distress of the patients. Our impression was that the absence of the lead gown during the ultrasound, as well as the setting itself of the dark sonography room, concurred in reducing the discomfort of the babies during the examination.

Posterior sagittal anorectoplasty (PSARP) was performed in all cases with no complications and intraoperative findings were equally concordant with the imaging studies. Postoperative course was uneventful in all cases.

## Discussion

At present the augmented-pressure distal colostogram is the gold standard for the preoperative assessment of ARMs without a visible fistula. 5 This examination, if adequately performed, allows the correct identification of the type of malformation, the location of the rectum, and presence of a recto-urinary fistula. 2 Errors in performing the preoperative imaging occur mostly due to inadequate application of pressure to the distal colostomy to overcome the muscle tone of the funnel-like striated muscle, which may induce to misinterpret the malformation as high and without fistula. In such cases, the risk of damaging adjacent structures such as the urethra, vas deferens, and seminal vesicles increases significantly. 6 Therefore, an attempt to reduce exposure to radiation, which is a known drawback of the distal colostogram, cannot be perpetuated at the cost of sacrificing the ground principles of this investigation. For this reason, MRI-fistulograms, 7 although potentially useful in combining the evaluation of the anatomy of the ARMs and the screening of associated malformations such as presacral masses and sacral ratio, 8 are not sufficiently reliable and have not gained space in the preoperative assessment of the ARMs.

Transperineal high-resolution ultrasound with 3D tomographic reconstruction can on the other hand be performed dynamically, keeping the same fundamental principles of the distal colostogram and assuring the application of adequate pressure to visualize the fistula. It also offers the advantage of a quick bedside testing method. Based on this premise, replacing the fluoroscopic examination with a sonography seems possible with sufficient experience, most likely after a supervised run-in phase with both methods performed and re-evaluated systematically. Other than the absence of radiation exposure, ultrasound offers a more comfortable setting both for the babies and for their families. Furthermore, high resolution transperineal ultrasound can detect presacral masses, thus further allowing the screening for Currarino syndrome, possibly replacing MRI in this context in newborns. 9

As for the evaluation of the relationship between the rectum and the bony structures of the pelvis, which is crucial for the choice of surgical approach, this appears possible in the sonographic evaluation since both the sacrum and the pubic symphysis are clearly visible in the images ( Fig. 1B, C ). Although promising, reliability needs to be assessed through a prospective trial before conclusions can be drawn in this regard.

Further studies with a higher number of patients, broader spectrum of disease (ARM with no fistula or with bladder-neck fistulas), and different age groups as well as blinded investigators are necessary to evaluate the reliability of high-resolution transperineal ultrasound with 3D tomographic reconstruction in the diagnosis of ARM.

Still this new technology merits attention and has the potential to drastically change diagnostic algorithms in ARMs.

## Conclusion

Based on our promising results, we envision high resolution transperineal ultrasound with 3D tomographic reconstruction to hold the potential to replace the distal colostogram at tertiary referral centers.

## Notes

New Insights and the Importance for the Pediatric Surgeon High-resolution transperineal ultrasound with 3D reconstruction seems to have the potential to replace the augmented pressure distal colostogram in anorectal malformations (ARM). This would allow a radiation-free assessment of the patients prior to the reconstructive surgery, drastically changing the current diagnostic algorithms in ARM.

## References

[1] Wood R J Levitt M A Anorectal malformations Clin Colon Rectal Surg 2018 31 02 61 70 29487488 10.1055/s-0037-1609020 PMC5825858

[2] Halleran D R Ahmad H Bates D G Vilanova-Sanchez A Wood R J Levitt M A A call to ARMs: accurate identification of the anatomy of the rectourethral fistula in anorectal malformations J Pediatr Surg 2019 54 08 1708 1710 31076157 10.1016/j.jpedsurg.2019.04.010

[3] Bischoff A Levitt M A Peña A Update on the management of anorectal malformations Pediatr Surg Int 2013 29 09 899 904 23913263 10.1007/s00383-013-3355-z

[4] Abdalla W MA De La Torre L The high pressure distal colostogram in anorectal malformations: technique and pitfalls J Pediatr Surg 2017 52 07 1207 1209 28381335 10.1016/j.jpedsurg.2017.03.050

[5] Kraus S J Levitt M A Peña A Augmented-pressure distal colostogram: the most important diagnostic tool for planning definitive surgical repair of anorectal malformations in boys Pediatr Radiol 2018 48 02 258 269 28840291 10.1007/s00247-017-3962-2

[6] Bischoff A Bealer J Wilcox D T Peña A Error traps and culture of safety in anorectal malformations Semin Pediatr Surg 2019 28 03 131 134 31171146 10.1053/j.sempedsurg.2019.04.016

[7] Kavalcova L Skaba R Kyncl M Rouskova B Prochazka A The diagnostic value of MRI fistulogram and MRI distal colostogram in patients with anorectal malformations J Pediatr Surg 2013 48 08 1806 1809 23932626 10.1016/j.jpedsurg.2013.06.006

[8] Krois W Palmisani F Gröpel P Assessment of sacral ratio in patients with anorectal malformations: Can magnetic resonance imaging replace conventional radiograph? J Pediatr Surg 2021 56 11 1993 1997 33485613 10.1016/j.jpedsurg.2021.01.013

[9] Köchling J Pistor G Märzhäuser Brands S Nasir R Lanksch W R The Currarino syndrome–hereditary transmitted syndrome of anorectal, sacral and presacral anomalies. Case report and review of the literature Eur J Pediatr Surg 1996 6 02 114 119 8740138 10.1055/s-2008-1066487

